# Structural and Electrical Response of Emerging Memories Exposed to Heavy Ion Radiation

**DOI:** 10.1021/acsnano.2c04841

**Published:** 2022-09-16

**Authors:** Tobias Vogel, Alexander Zintler, Nico Kaiser, Nicolas Guillaume, Gauthier Lefèvre, Maximilian Lederer, Anna Lisa Serra, Eszter Piros, Taewook Kim, Philipp Schreyer, Robert Winkler, Déspina Nasiou, Ricardo Revello Olivo, Tarek Ali, David Lehninger, Alexey Arzumanov, Christelle Charpin-Nicolle, Guillaume Bourgeois, Laurent Grenouillet, Marie-Claire Cyrille, Gabriele Navarro, Konrad Seidel, Thomas Kämpfe, Stefan Petzold, Christina Trautmann, Leopoldo Molina-Luna, Lambert Alff

**Affiliations:** † Advanced Thin Film Technology Division, Institute of Materials Science, TU Darmstadt, Alarich-Weiss-Str. 2, 64287 Darmstadt, Germany; ‡ Advanced Electron Microscopy Division, Institute of Materials Science, TU Darmstadt, Alarich-Weiss-Str. 2, 64287 Darmstadt, Germany; § CEA, LETI, Univ. Grenoble Alpes, 38000 Grenoble, France; ∥ CNRS-LTM, CEA, LETI, 38054 Grenoble, France; ⊥ Fraunhofer IMPS, Center Nanoelectronic Technologies (CNT), 01109 Dresden, Germany; # GSI Helmholtzzentrum fuer Schwerionenforschung, 64291 Darmstadt, Germany; % Institute of Materials Science, TU Darmstadt, 64287 Darmstadt, Germany

**Keywords:** ferroelectric random-access memory, hafnium oxide, phase-change memory, phase transitions, resistive random-access memory, swift heavy ions, 4D-scanning transmission electron microscopy, automated crystal orientation mapping

## Abstract

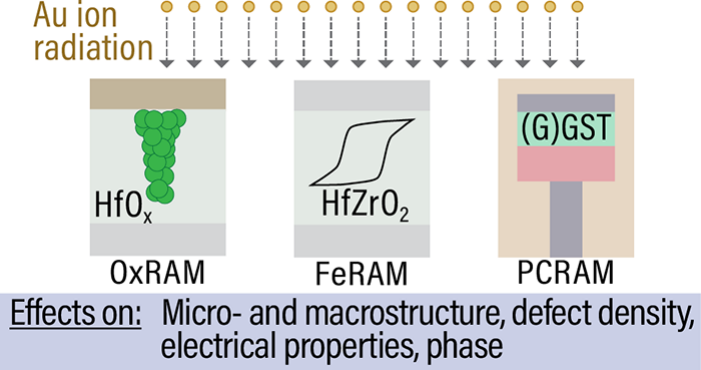

Hafnium oxide- and GeSbTe-based functional layers are promising candidates in material systems for emerging memory technologies. They are also discussed as contenders for radiation-harsh environment applications. Testing the resilience against ion radiation is of high importance to identify materials that are feasible for future applications of emerging memory technologies like oxide-based, ferroelectric, and phase-change random-access memory. Induced changes of the crystalline and microscopic structure have to be considered as they are directly related to the memory states and failure mechanisms of the emerging memory technologies. Therefore, we present heavy ion irradiation-induced effects in emerging memories based on different memory materials, in particular, HfO_2_-, HfZrO_2_-, as well as GeSbTe-based thin films. This study reveals that the initial crystallinity, composition, and microstructure of the memory materials have a fundamental influence on their interaction with Au swift heavy ions. With this, we provide a test protocol for irradiation experiments of hafnium oxide- and GeSbTe-based emerging memories, combining structural investigations by X-ray diffraction on a macroscopic, scanning transmission electron microscopy on a microscopic scale, and electrical characterization of real devices. Such fundamental studies can be also of importance for future applications, considering the transition of digital to analog memories with a multitude of resistance states.

Hafnium oxide- (HfO_*x*_) and germanium–antimony-telluride (GeSbTe or GST)-based functional materials face increasing attention due to their promising properties when acting as functional layers in emerging nonvolatile memory applications.^1−5^ Oxide-based random-access memory (OxRAM) and ferroelectric-random-access memory (FeRAM) can both be based on HfO_*x*_.^6−9^ GeSbTe-based layers are the most prominent active materials used for phase-change memory (PCM or PCRAM), which together with OxRAM belongs to the class of resistive random-access memory (RRAM). The memory in OxRAM is stored in distinct electrical resistance states, namely a high resistance state (HRS) and a low resistance state (LRS), accessible by formation and rupturing of conductive oxygen vacancy filaments.^10,11^ In FeRAM, different memory states are achieved by setting distinct electrical polarization states of a polar crystalline structure.^6,7^ The principle of memory storage in PCM is based on a reversible transition between an ordered crystalline (LRS) and a disordered amorphous phase (HRS) of a chalcogenide material. This phase change is controlled by applying heat via controlled voltage pulses to the phase-change layer. The most-promising chalcogenide-based material for PCM is Ge_2_Sb_2_Te_5_ (GST). By adding Ge to GST, a Ge-rich GST alloy (GGST) can be created, showing good stability of the amorphous phase at higher temperatures compared to GST. This makes GGST a very promising candidate for automotive applications.^12−14^ PCRAM is often considered the most mature RRAM technology for application due to its already proven manufacturability, meeting the strict requirements of embedded applications.^13^ In general, a fundamental knowledge about radiation hardness and corresponding failure mechanisms in nonvolatile memories is needed, in particular, e.g., aerospace applications. Compared to charge-based flash technologies, the underlying mechanisms of these emerging memories are strongly dependent on defects and on the crystal structure of the active material. Enhanced resilience toward ionizing radiation has been demonstrated, making them promising candidates for radiation-hard memories. Several studies on the radiation-hardness of hafnium oxide-^15−22^ and GeSbTe-based^22−29^ material systems have been reported. These cover proton^16,20,30−34^ and heavy ion irradiations^19,32,35−37^ of memristive devices for, e.g., oxide-based RRAM and FeRAM^33,34^ applications. For PCM, studies have also discussed beam-induced changes depending on the structure of devices and arrays,^22−29^ often reporting secondary failures due to the degradation of the CMOS transistors used in the electric circuits. The overall increasing interest in radiation effects and hardness of memories is discussed in recent publications by Marinella^38^ and Fleetwood.^39^ In general, ion beams can also be applied in a constructive manner to intentionally tailor properties of functional oxide layers.^40,41^ The detailed mechanisms of the interaction processes of ions with matter are not yet fully understood, still in oxide-based RRAM and FeRAM the creation of defects, most likely oxygen vacancies, is involved. Already small changes introduced by irradiation can influence the resistive (switching) behavior.^42−44^ Another important phenomenon is related to phase transitions which can occur when a certain energy loss threshold and fluence is exceeded. A prominent example is the phase transition of initially monoclinic HfO_2_ grains to the tetragonal phase above an electronic energy loss threshold of about 18 keV/nm as described by A. Benyagoub.^45−48^ Such phase transitions are often directly related to the formation of tracks^49^ and modeled by an inelastic thermal spike.^50,51^ The phase transition mechanism for HfO_2_ is described by a double hit process, where a first ion impact creates oxygen defects and a second ion hit of the defective track zone leads to a macroscopically detectable phase transition. Information how thin hafnium-oxide based films respond to energetic ions is rather scarce and inconclusive.^52−54^ In highly textured hafnium oxide films, an oxygen defect-induced phase transition was reported.^55^ In contrast to the radiation-induced phase transition from the monoclinic to the tetragonal structure, we recently identified the oxygen-deficient HfO_*x*_ phase as a defect-stabilized variant of the monoclinic structure with a slightly rhombohedral distorted cubic symmetry (low-temperature phase of cubic HfO_*x*_, named LTP *c*-HfO_2–*x*_) in as-grown hafnium oxide films.^56^ Regarding such effects occurring on a microstructural scale, especially transmission electron microscopy (TEM) has been proven to be a very powerful tool to investigate structural changes on a micrometer or nanometer scale.^54,57−59^ Structural changes become especially relevant in ferroelectric and phase-change memory, as their information storage is based on the crystallinity of the active layer. Its evolution is essential for memory cell functionality. In literature, ion irradiation-induced phase changes were reported,^60,61^ including effects on the crystallization of amorphous Ge_2_Sb_2_Te_5_^61^ and a high stability of the amorphous state.^62^

Results available to date clearly indicate that not only changes on the physical and electrical properties of irradiated memory materials play an important role, but also especially associated effects on the crystallinity, the microstructure, and additionally on the local composition have to be considered. Such effects are barely considered, but they are relevant for a detailed understanding of general mechanisms in emerging memory technologies. In this paper, we concentrate on the irradiation of technologically important HfO_*x*_- and GeSbTe-based emerging memory materials of different composition with high energy swift heavy ions and discuss the experimental data in the context of initial crystallinity, initial microstructure and irradiation-induced changes. By combining conventional methods such as X-ray diffraction (XRD) that is probing a larger volume, with methods of high spatial resolution like scanning transmission electron microscopy (STEM), an observation of the response of the functional thin films on a local level is possible. Our work provides an exemplary test protocol for heavy ion irradiation experiments and investigations performed on HfO_*x*_- and GeSbTe-based emerging memory materials and devices, combining structural investigations and electrical characterization of real devices. The established methodology allows two key advancements, (i) development strategies of radiation-hard memory for applications in radiation-harsh (such as space or accelerator) environments and (ii) improved understanding of the basic mechanisms of irradiation effects on memory materials. Although this work is not focusing on neuromorphic properties relying on analogue resistive values, the here reported results will be also a solid base for future investigations of memristive devices in neural networks.

## Results and Discussion

### Hafnium Oxide- and GeSbTe-Based Sample Series

**Figure 1:**
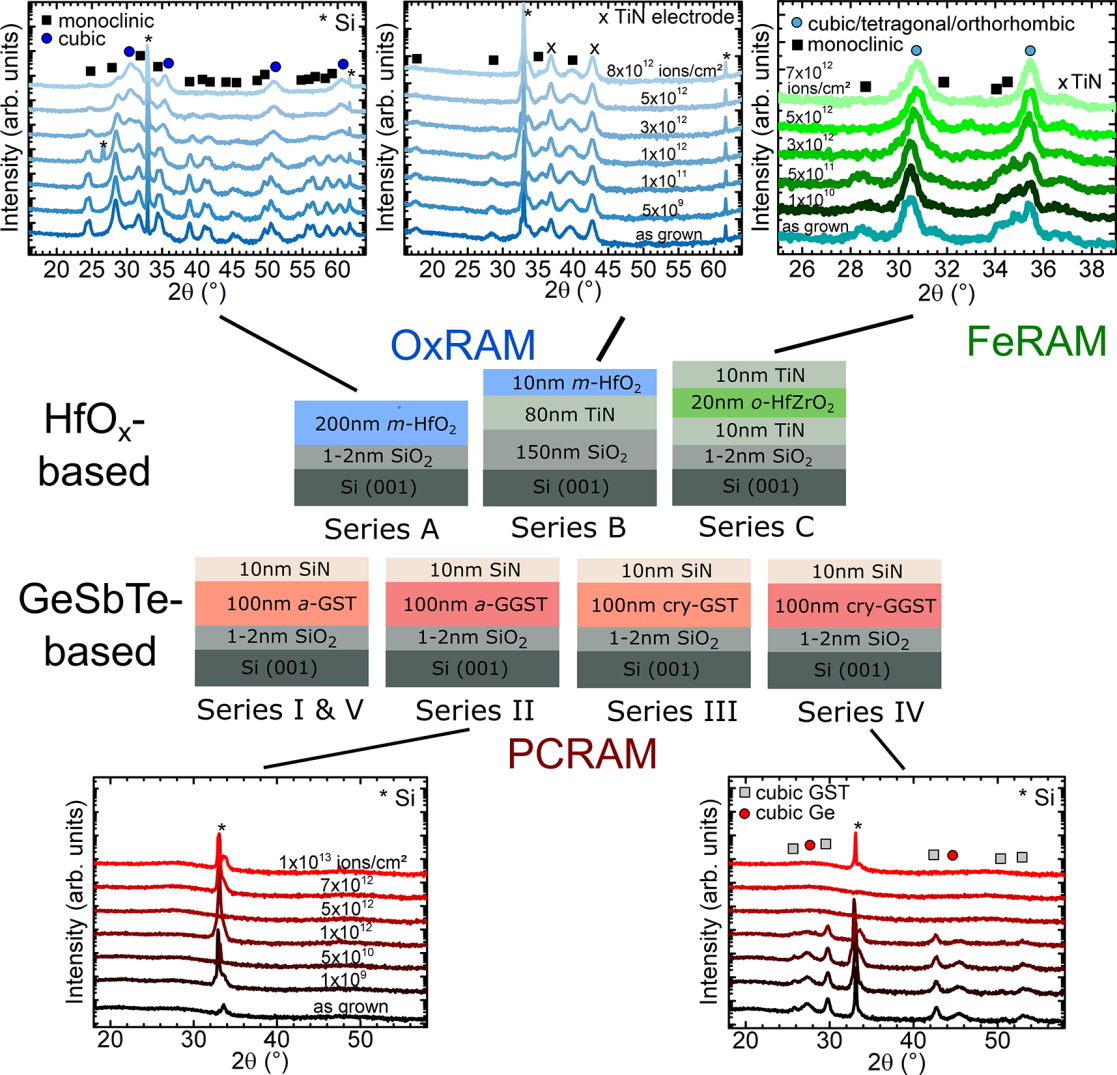
Schematic overview of sample series A–C containing HfO_*x*_-based layers (HfO_2_ and HfZrO_2_) and sample series I–V containing GeSbTe-based layers (GST and GGST). Representative XRD patterns before and after ion irradiation show major structural changes.

Sample series of hafnium oxide-based layers (HfO_*x*_-based) (indexed as A, B, and C) and of GeSbTe-based layers (indexed in Roman numbers: I, II, III, IV, and V) were investigated. A schematic overview of these sample is presented in Figure 1 together with exemplary XRD patterns before and after ion irradiations with 1.635 GeV Au ions of various fluences (5 × 10^9^ to 8 × 10^12^ ions/cm^2^). Those are meant as a first overview to give a short insight in the major structural differences. A short description of the investigated samples is given in the following. The results are discussed in detail later in the corresponding discussion parts.

Series A: 200 nm thick films of stoichiometric monoclinic hafnium oxide (*m*-HfO_2_) (space group *P*21/*a*; ICDD: 00-034-0104) grown on SiO_2_/Si substrates by utilizing electron beam evaporation and an oxygen plasma

Series B: 10 nm thin *m*-HfO_2_ films grown by atomic layer deposition (ALD) on top of TiN bottom electrodes on SiO_2_/Si substrates

Series C: 20 nm thin hafnium zirconium oxide layers (Hf_0.5_Zr_0.5_O_2_, labeled as HfZrO_2_) sandwiched between TiN electrodes on SiO_2_/Si substrates. HfZrO_2_ has ferroelectric properties due to the orthorhombic phase (space group *Pca*2_1_; ICDD: 04-005-5597).

Structural XRD investigations were performed and results are directly connected to results obtained by STEM (4D-STEM, automated crystal orientation mapping (ACOM)) (series A vs B) and the electrical behavior of OxRAM (10 nm HfO_2_-based) and FeRAM (ferroelectric stacks, series C) before and after irradiation.

Sample series I–V include 100 nm thick films of GST and Ge-rich GeSbTe (GGST) of amorphous (*a*-GST, *a*-GGST) and crystalline (*cry*-GST, *cry*-GGST) structure on SiO_2_/Si substrates. A SiN capping layer is placed on top. Crystalline films were achieved by postdeposition annealing. Again, structural results obtained by XRD and STEM are related to electrical results of PCRAM devices exposed to Au heavy ions. More detailed material information and the most important sample parameters are provided in the Methods and in Table 1.

**Table 1:** Important Characteristics of the Hafnium Oxide- and GeSbTe-Based Sample Series

sample series	A	B	C	I	II	III	IV	V
Functional layer	HfO_2_	HfO_2_	Hf_0.5_Zr_0.5_O_2_	GST	GGST	GST	GGST	GST
Growth technique	PVD/MBE	ALD	ALD	GST – single target sputtering/GGST – cosputtering				
Growth temperature	300 °C	300 °C	300 °C	60 °C	60 °C	60 °C	60 °C	60 °C
Layer thickness	200 nm	10 nm	20 nm	100 nm	100 nm	100 nm	100 nm	100 nm
Substrate^a^	SiO_2_/Si	TiN/SiO_2_/Si	TiN/SiO_2_/Si	SiO_2_/Si	SiO_2_/Si	SiO_2_/Si	SiO_2_/Si	SiO_2_/Si
Special information	Hf e-beam evaporation and oxygen plasma	HfCl_4_ and H_2_O	HfCl_4_ and ZrCl_4_ and O_3_ postannealed 400 °C/1 h	amorphous layer		postannealing for 15 min @ 450 °C for crystallization		amorphous layer
Irradiation conditions	Energy: 8.3 MeV/u Au ≙ 1.635 GeV Flux: 5 × 10^8^ ions/cm^2^ s Fluence range: 5 × 10^9^ – 8 × 10^12^ ions/cm^2^ (HfO_2_-based stacks) and 1 × 10^13^ ions/cm^2^ (GeSbTe-based stacks)							

a The term “Substrate” includes all layers of material on which the respective active layers were grown.

### Irradiation-Induced Phase Transitions in Hafnium Oxide and Correlated Electrical OxRAM Device Properties

**Figure 2:**
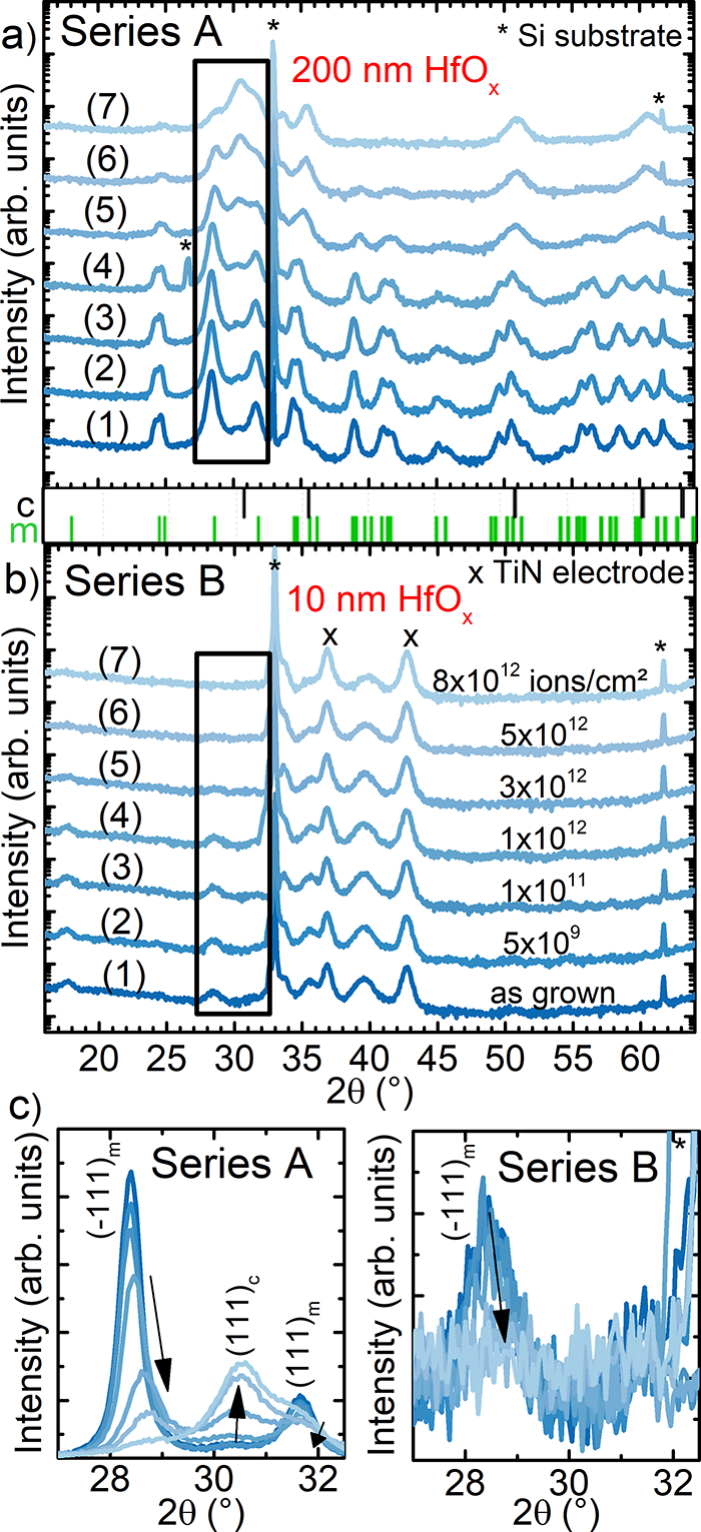
XRD patterns of HfO_2_ films before (as grown) and after irradiation. (a) XRD patterns of 200 nm thick HfO_*x*_ films on SiO_2_/Si substrates (series A), indicating a phase transition from the monoclinic (green reference pattern) to a cubic phase (black reference pattern) of hafnium oxide after irradiation. (b) XRD patterns of 10 nm thick HfO_*x*_ films on TiN/SiO_2_/Si (series B), revealing a drop of intensity without a directly visible crystalline-to-crystalline phase transition. Si reflections are marked with a *. (c) Detailed views of the XRD patterns of (a) and (b) in the region of the (−111)_*m*_ reflection. Reflections of the monoclinic and cubic phase are marked with m and c, respectively. Black arrows are used to illustrate trends occurring with increasing fluence.

**Figure 3:**
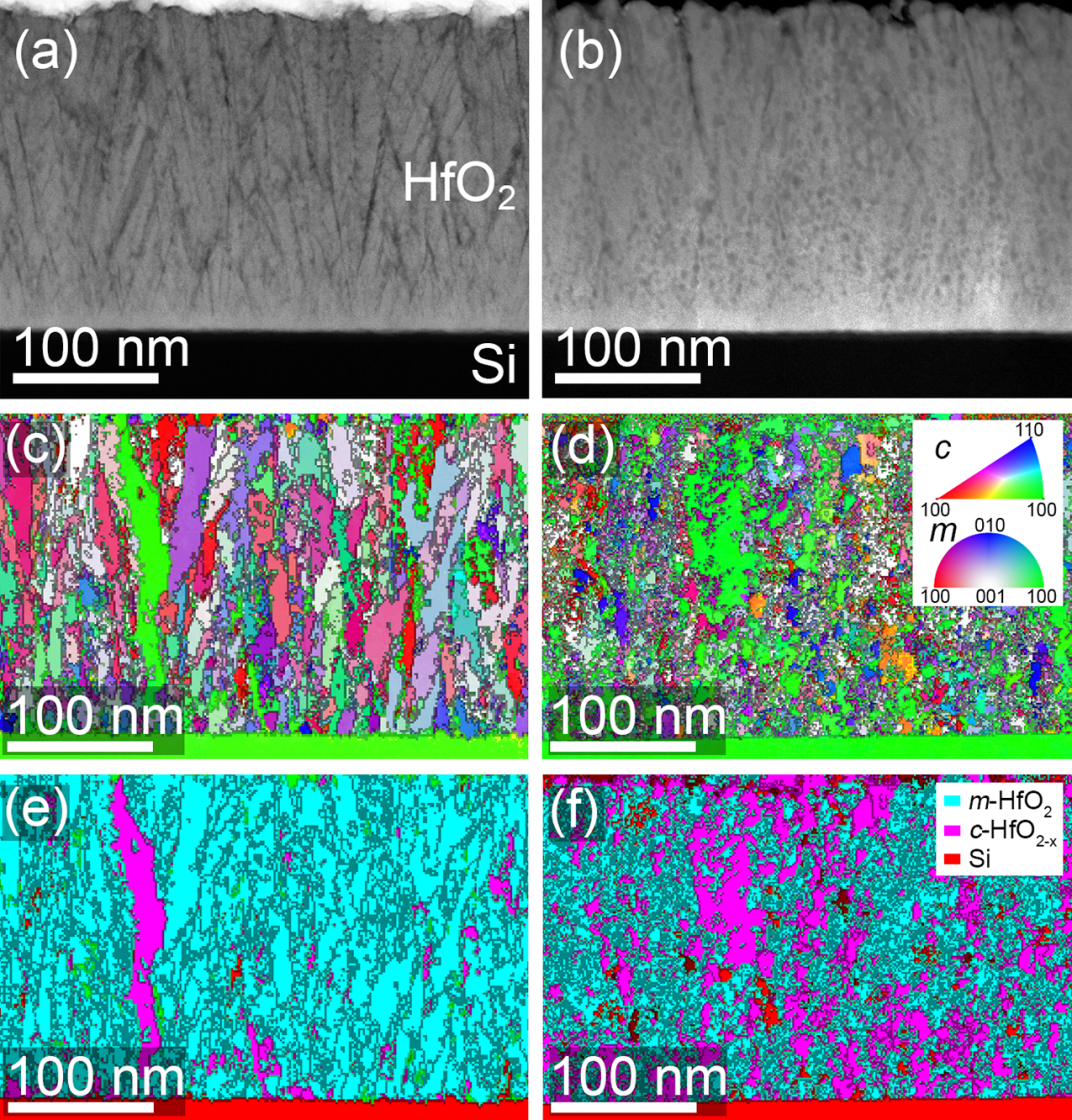
Microstructural investigations of series A before (a,c,e) and after irradiation with 5 × 10^12^ ions/cm^2^ (b,d,f). (a) HAADF-STEM image of the unirradiated reference HfO_2_/SiO_2_/Si stack with large columnar grains visible. (b) HAADF-STEM image of an irradiated sample (5 × 10^12^ ions/cm^2^) (c) ACOM orientation map of an as-grown film, each color represents an orientation. (d) ACOM orientation map of an irradiated sample (5 × 10^12^ ions/cm^2^), including a color wheel for the cubic and monoclinic structure. A significant grain fragmentation is identified. (e) ACOM phase map of a reference sample (as-grown film) with high fraction of indexed *m*-HfO_2_ (gray overlays indicate boundaries of >10° misorientation and phase boundaries). (f) ACOM phase map of an irradiated sample (5 × 10^12^ ions/cm^2^) with a higher phase fraction of a cubic phase (9% and 42% before and after irradiation, respectively). For both ACOM maps the same image processing and template matching parameters were used.

XRD patterns of as-grown and irradiated initially monoclinic hafnium oxide (*m*-HfO_2_) films (series A and B) are shown in Figure 2. As-grown 200 nm thick films of series A (Figure 2a), corresponding to a HfO_2_ layer grown by oxygen plasma assisted electron beam evaporation on a SiO_2_/Si substrate, show reflections of the monoclinic space group *P*21/*a*, (ICDD: 00-034-0104). The growth of thick films on top of an oxidized Si substrate (native SiO_2_ layer) leads to the formation of a polycrystalline layer, where the reflection with the highest intensity at 2θ ≈ 28.4° corresponds to the (−111) lattice plane. The irradiation with 1.635 GeV Au ions induces a crystalline-to-crystalline phase transition that starts at a fluence above 1 × 10^12^ ions/cm^2^ and progresses with increasing fluence. At a kinetic energy of 1.635 GeV, the Au ions penetrate through the whole film (ion range ≫1 μm) and stop in the substrate. According to the TRIM-2010 code,^63^ the electronic energy loss of the ions in the HfO_2_ films (density 9.68 g/cm^2^^64^) is 53 keV/nm. The slowing down process is clearly dominated by electronic interactions, and the nuclear energy loss is only about 69 eV/nm, resulting in a low number of displacements per atom (dpa) of about 4.4 × 10^–4^ (determined by full cascade TRIM calculations). The observed phase transition is in agreement with earlier reports^47^ stating that the transition requires an electronic energy loss of at least 18 keV/nm and a fluence high enough to yield overlapping tracks. Both mandatory conditions for the defect-induced phase transition were fulfilled in our experiment. According to earlier irradiations of thin films, the number of oxygen-defects rises with increasing ion fluence, resulting in the creation of HfO_*x*_ suboxides (oxygen-deficient hafnium oxide).^55^ The crystalline phase induced by the ion irradiation was so far assigned to a tetragonal phase.^47,55^ However, we note that the cubic, tetragonal, and several orthorhombic phases in polymorphic hafnium oxide are difficult to distinguish by XRD. We thus complemented XRD by analyzing nanobeam electron diffraction (NBED) patterns obtained from 4D-STEM investigations, which allows us to assign the achieved defect-stabilized phase to the low temperature phase of cubic hafnium oxide (LTP *c*-HfO_2–*x*;_^56^ space group: distorted *Fm*3̅*m*, ICDD: 04-011-9018), recently described in detail by Kaiser et al.^56^ This is validated by matching simulated nanobeam electron diffraction (NBED) patterns for the suggested phase to the experimental NBED patterns acquired at the nanocrystals in the irradiated films (Figure 3), which is a significant extension to the so far reported results on irradiated hafnium oxide. Figure 2b presents the XRD patterns of 10 nm thin monoclinic HfO_*x*_ films grown on TiN/SiO_2_/Si (series B) before and after ion irradiation. Such very thin oxide layers are of great relevance for real industrial RRAM applications with the conducting TiN layer serving as the bottom electrode. The important role of the film thickness and the increasing difficulties of test scenarios when down-scaling electronics was recently discussed by Fleetwood.^39^ This problem is reflected in the rather low intensity of the reflections in the XRD patterns of the thinner HfO_*x*_ films of series B due to the reduced crystalline volume. At a fluence of around 3 × 10^12^ ions/cm^2^, the initially prominent reflection of the (−111) lattice plane (at 2θ ≈ 28.45°) considerably reduces in intensity, without showing evidence for a transition to the LTP *c-*HfO_2-x_ phase. We will show that this is not simply the result of the overall reduced intensity and signal-to-noise ratio due to the lower crystalline quality as compared to series A. A more detailed representation of the relevant 2θ range is given in Figure 2 c), were the transition from monoclinic ((−111) and (111) reflections) to a cubic phase ((111) reflection) is seen for series A, while only a reduction of the (−111) reflection intensity is observed for series B. The Si substrate reflection observed at 2θ ≈ 32–34° corresponds to the forbidden (200) reflection (Umweganregung). Its intensity and shape are dependent on the Φ-position during XRD (planar horizontal rotation of the sample),^65^ which does not affect the results obtained for layers grown on top.

This phase transition is accompanied by a shift and broadening of the reflections. A shift can be considered as an indication for an increased number of induced defects in the defect-stabilized crystalline structure. A reflection broadening can be associated on the one hand with a possibly reduced average grain size and on the other hand to an increased number of defects in the crystalline lattice, leading to a larger variation of the lattice planes. These phenomena are directly comparable to XRD results obtained from nonirradiated oxygen-deficient HfO_*x*_ layers in the literature.^56^ As a possibility to explain the reduction of the reflection intensity, amorphization or grain fragmentation of crystalline grains has to be considered as well. Here, XRD does not allow us to clarify the precise nature of the induced changes on the nm scale, and possible amorphization effects can only be resolved via a spatially resolved data set. Therefore, STEM investigations have been performed to correlate the direct effect of ion irradiation on the microstructure. As a gradual change of the structural changes in the XRD patterns is observed and the highest chance of detecting a high amount of a crystalline cubic phase (after the phase transition) is given at high fluences above 1 × 10^12^ to 3 × 10^12^ ions/cm^2^, samples exposed to 5 × 10^12^ ions/cm^2^ have been chosen for the STEM investigations from both series A and B (samples labeled (6) in Figure 2). Nonirradiated samples with a high content of the cubic phase served as reference (samples labeled (1)). Figure 3 shows cross sections of 200 nm thick HfO_*x*_ films of series A before (as grown) and after irradiation with a fluence of 5 × 10^12^ ions/cm^2^. Panels a and b show the samples in high-angle annular dark-field (HAADF) contrast. Before irradiation, the grain boundaries (dark lines in panel a) are clearly visible, while the image of the irradiated sample (panel b) is rather diffuse with only a small number of dark lines. To better identify the granular microstructure for both samples, automated crystal orientation maps (ACOM) are generated. Therefore, the electron diffraction information on the scanning precession electron diffraction (SPED) data set is processed in a template matching routine, that assigns probability values for a list of user defined templates for each real space position (for each pixel in the map). Here, motivated by the findings in XRD and earlier work,^56^ we applied the *m*-phase of HfO_2_ and the low-temperature *c*-phase of HfO_2–*x*_ as input. Matching the templates during ACOM extracts two types of information from the SPED map. First, parts c and d of Figure 3 show the orientations of all grains in the sampled area, and each color corresponds to an individual orientation of the sampled grain (inverse pole figure (IPF) colored). The pristine sample consists of (columnar) grains of several tens of nm to more than 100 nm length, which are reduced to sizes below 10–30 nm for the irradiated sample. A significant grain fragmentation is identified. Parts e and f of Figure 3 show the ACOM map with the color code indicating the matched phase in the data set. Second, the information about the ACOM attributed phase is shown in Figure 3 e,f. The localized identification of the monoclinic and cubic phase before (Figure 3e) and after irradiation (Figure 3f) gives a detailed view of the irradiation-induced phase transition. Plotting the phase information extracted from the SPED data set emphasizes this phase transition with a visible phase separation and directly illustrates how the cubic phase is formed in a nanocrystalline manner. Approximate phase fractions are about 91% monoclinic to 9% cubic phase for the nonirradiated (as grown) reference and 58% monoclinic to 42% cubic phase after exposure of 5 × 10^12^ ions/cm^2^. A slight underestimation with an approximate uncertainty of 10% in the case of the matching is possible. To confirm the validity of the recognized phases in the ACOM data set, the available nanobeam electron diffraction (NBED) patterns have been averaged for the classes created in the template matching routine. Rotationally averaging and integrating the averaged electron diffraction patterns for the *m*-HfO_2_ and LTP *c*-HfO_2–*x*_ phases clearly indicates that it was possible to discern the two phases by this method. Supporting Information 1 shows the converted 2θ plots of the position averaged NBED (PANBED) diffraction data compared to the corresponding XRD 2θ scans. Limited by the used beam convergence in the SPED experiment (5 mrad at 200 kV electrons equaling 17 °Cu K_α_ radiation), the observed phase transition is confirmed from the high spatial resolution SPED data set. More information on the workflow of integrating a 4D-STEM SPED data set is provided in an open repository including the raw data.^66^

**Figure 4:**
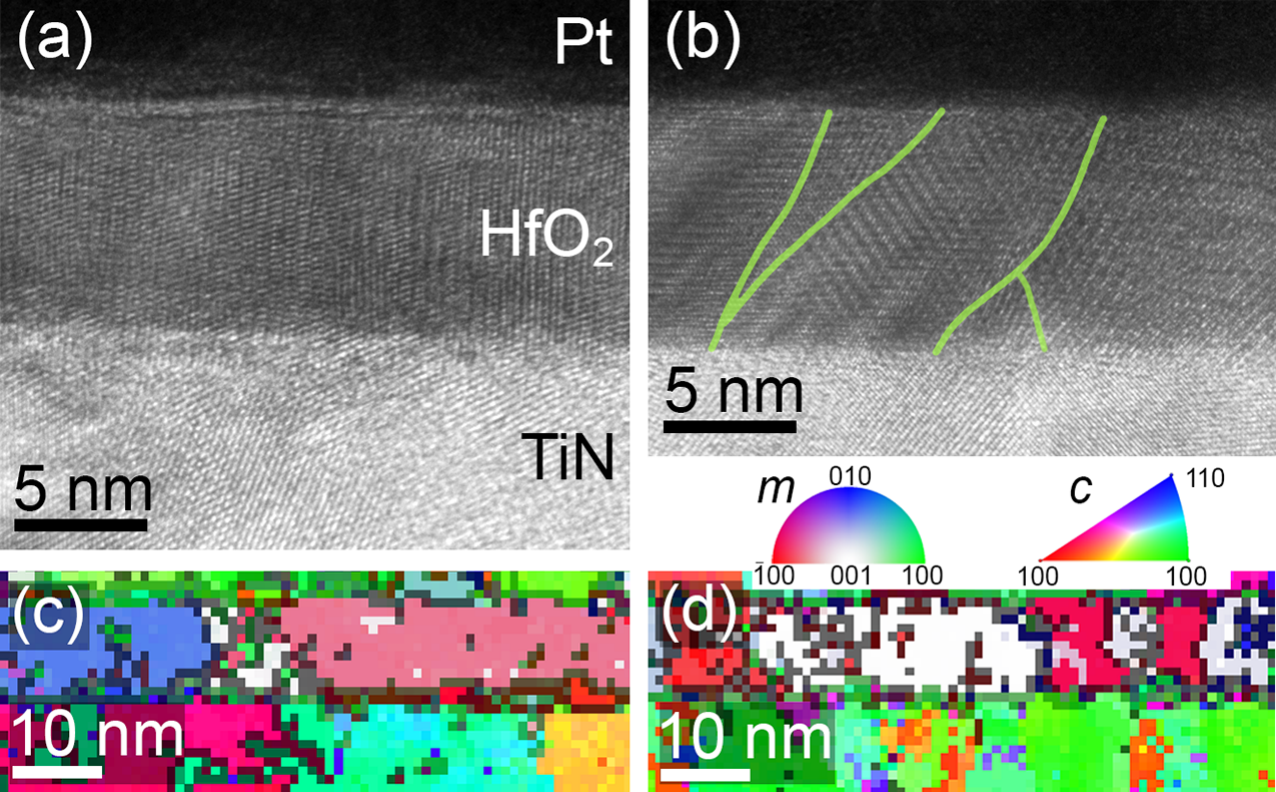
Microstructural investigations of series B. (a) HRTEM image of the reference (as grown) Pt/HfO_2_/TiN stack with one single grain visible in the field of view. (b) Representative HRTEM image of an irradiated sample (5 × 10^12^ ions/cm^2^) with significantly smaller grains. Grain boundaries are highlighted by the green lines. (c) ACOM orientation map of the reference (as grown) sample, where each color represents a different orientation (gray indicates misorientation (>20°) and phase boundaries). (d) ACOM orientation map of an irradiated sample (5 × 10^12^ ions/cm^2^). Note that the electrode layers (TiN and Pt) are also included in the map.

For the 10 nm thin as-grown HfO_*x*_ films of series B, similar phase orientation investigations revealed grains vertically at least as large as the film thickness and horizontally larger than the image of Figure 4a. The microstructure resembles the initial 10 nm of the 200 nm thick films shown in Figure 3c,d, accompanied by a slightly lower texture. After irradiation at a fluence of 5 × 10^12^ ions/cm^2^, similar as for the thick film, a fragmentation of the hafnium oxide grains is observed, which is manifested by a lateral size reduction (Figure 4b,d). It is important to mention that for the films shown in Figures 3 and 4 (series A vs series B) the microstructural investigations reveal crystalline grains after irradiation, further confirming a pure crystalline-to-crystalline phase transformation with no traces of amorphization. The vanishing diffraction intensities of the monoclinic reflections in the XRD patterns (Figure 2b) for the thinner films of series B at high fluences (>1 × 10^12^ ions/cm^2^) can therefore be solely explained by grain fragmentation as confirmed by the presented STEM results. For the 10 nm thin HfO_*x*_ films, the STEM information is extremely helpful because the interaction volume of coherent crystalline regions of one orientation is not sufficient to obtain resolvable intensities in the XRD patterns. Although the low available volume (real space pixels) for pattern matching of the phases did not yield in a representative phase volume ratio, the results obtained for 200 nm thick HfO_*x*_ films (Figures 2a and 3c,d) verify the pure crystalline-to-crystalline phase transition behavior also in thin films (Figure 2b). Additionally, in films of both thicknesses, the ACOM maps show elongated grains with a the [−111] direction being present in all films before and after irradiation, which fully matches the observed changes in the XRD patterns. The ACOM results of irradiated HfO_*x*_ films combined with XRD results demonstrate the fully micro- and nanocrystalline nature of all films of series A and B. This suggests strongly a heavy ion irradiation-induced crystalline-to-crystalline phase transition accompanied by significant grain fragmentation, otherwise possibly falsely identified as amorphization.

**Figure 5:**
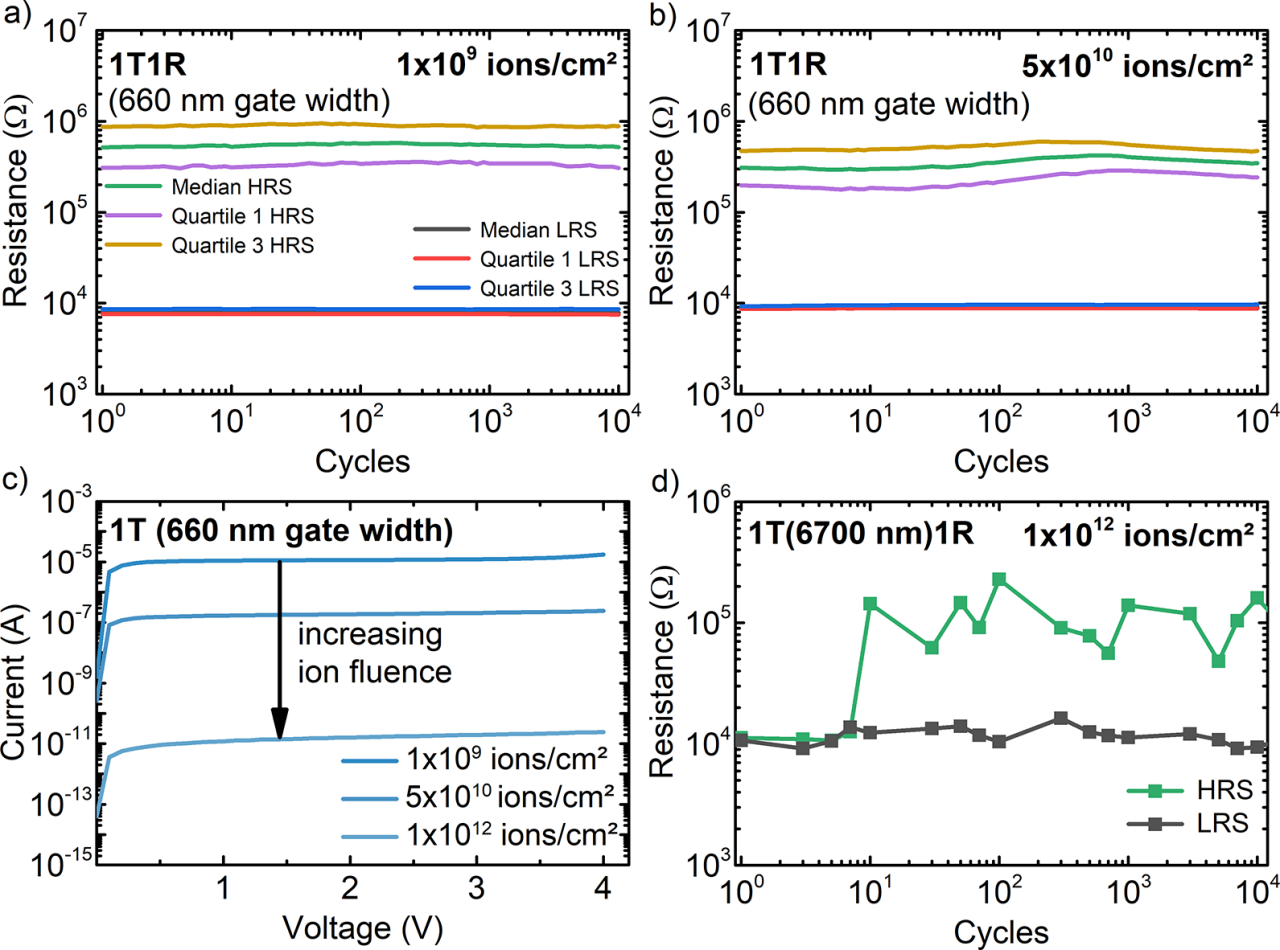
Functional tests of 1T1R arrays containing 10 nm thin HfO_2_ layers corresponding to series B. Resistance versus 10^4^ cycles for a 1T1R HfO_2_ sample containing 3072 devices after irradiation at a fluence of (a) 1 × 10^9^ ions/cm^2^ and (b) 5 × 10^10^ ions/cm^2^. (c) Current–voltage curves of 660 nm gate width transistors after irradiation with different fluences. (d) Exemplary 10^4^ cycles performed on a 1R cell of a 1T1R HfO_2_ containing device irradiated at a fluence of 1 × 10^12^ ions/cm^2^. Data presented at the RADECS 2020 conference; proceedings pending.^68^

The structural results can be directly correlated to the electrical behavior of hafnium oxide-based OxRAM devices. After irradiation with a fluence of 1 × 10^9^ ions/cm^2^, the integrated 1 transistor(660 nm gate width)-1 resistive memory element (1T1R) array is fully functional (Figure 5a) with no measurable irradiation-induced resistance changes. The corresponding memory window is maintained for at least 10^4^ cycles for more than 3000 measured devices, providing a good statistical certainty.^67^ The good performance is still preserved after irradiation at higher fluences of 5 × 10^10^ ions/cm^2^ (Figure 5b). At a fluence of 1 × 10^12^ ions/cm^2^ (see I–V characteristics in Figure 5c), a significant impact on the access transistor functionality was observed. This prevented an automatized access of the memory cells via the transistors (660 nm) for cells exposed to the highest fluence. Still, the memory cell (1R) of the sample exposed to 1 × 10^12^ ions/cm^2^ could be accessed by connecting a large gate width transistor (6700 nm) in series to provide a sufficiently high current. This memory cell first seemed to be stuck in the LRS, but after 10 cycles it recovered, probably due to a redistribution of oxygen vacancies by voltage cycling and showed resistive switching with two distinct states (HRS and LRS) for at least 10^4^ cycles. Overall, the memory cells are still fully functional, i.e., showing distinct memory states, after ion exposure to a fluence as high as 1 × 10^12^ ions/cm^2^.

The device size of about 160000 nm^2^ (1.6 × 10^–9^ cm^2^) is much smaller than the area of a full-sheet layer sample, resulting in a much lower number of ions impacting on each device. At a fluence of 1 × 10^12^ ions/cm^2^ each memory cell is hit in average by 1600 ions. Assuming a damage cross section of the ion tracks in HfO_2_ (48–74 nm^2^)^47,55^ and considering overlapping tracks, the areal track coverage at a fluence of 1 × 10^12^ ions/cm^2^ is between 38% and 52% (at a fluence of 8 × 10^12^ ions/cm^2^, and the corresponding numbers increase to 98% and 100%, respectively). It becomes obvious that the probability of track overlapping cannot be ignored. Still, the functionality of OxRAM devices that were exposed to 1 × 10^12^ ions/cm^2^ is still preserved. These results evidence that HfO_2_-based resistive memory devices are radiation hard memories. For even higher fluences, we expect that the electrical properties further change according to the increased cubic phase in the hafnium oxide layer. According to investigations on as-grown samples with the LTP *c*-HfO_2*-x*_ phase by Kaiser et al.,^56^ the device conductivity is expected to increase drastically. If a large amount of vacancies has been introduced and a larger amount of cubic phase has been formed, the memory cells will remain fully conducting (lost memory).

### Irradiation-Induced Phase Transitions in Doped Hafnium Oxide Ferroelectric Layers and Correlated FeRAM Device Properties

**Figure 6:**
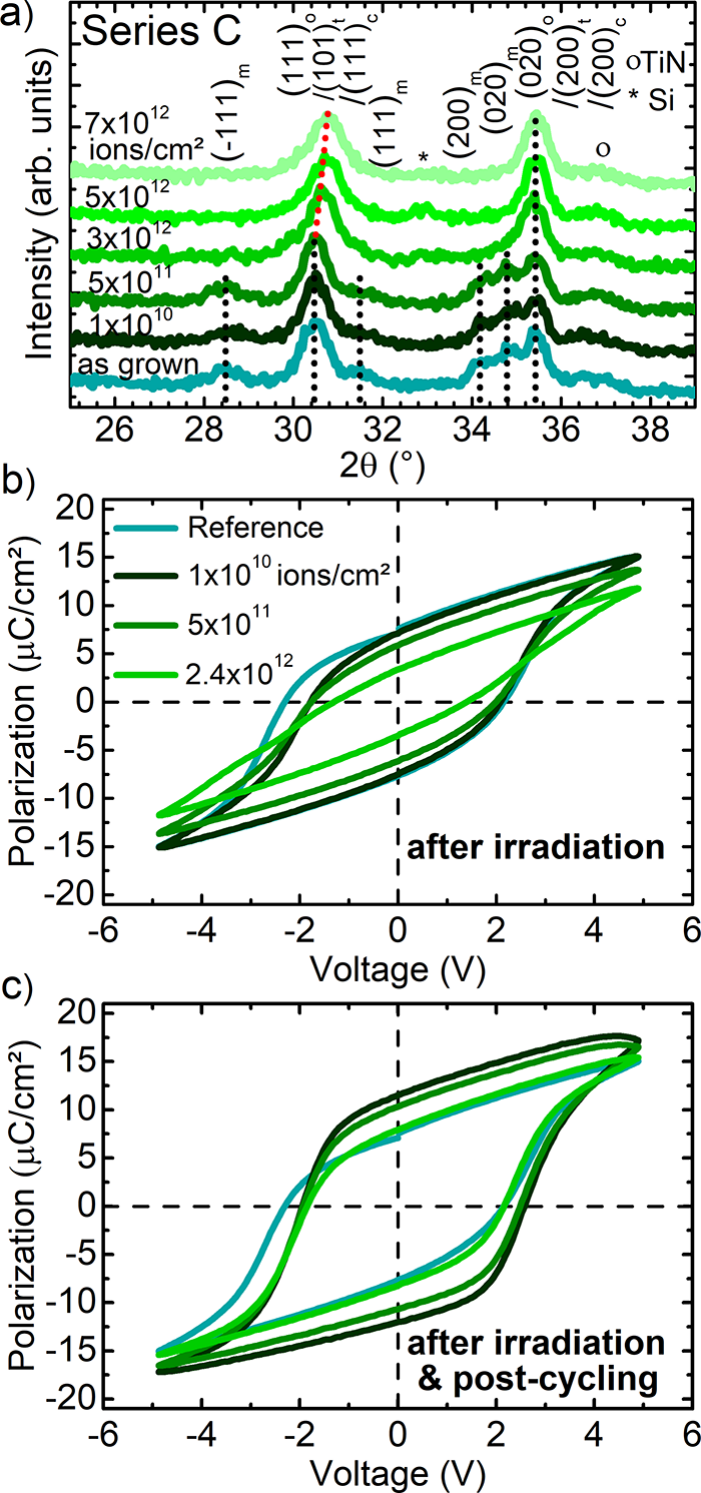
(a) XRD patterns of as-grown and irradiated ferroelectric HfZrO_2_ films (with TiN electrodes) of series C, revealing a phase transition from a polar orthorhombic to a nonpolar phase at fluences above 10^12^ ions/cm^2^. The forbidden Si (200) reflection is marked with a *; the TiN electrode reflections are marked with a circle. (b) Polarization as a function of applied voltage of as-grown unirradiated (black line) and irradiated stacks. (c) Recovery of the polarization-voltage properties after 10^4^ times postcycling of the irradiated samples compared to an as-grown sample (black line).

Similar to OxRAM, also FeRAM can be based on the HfO_*x*_ material system when proper elemental doping (e.g., Zr) or film stress is applied to stabilize the ferroelectric phase. Due to a different preparation for FeRAM cells (e.g., growth of amorphous Zr-doped HfO_*x*_ and postannealing leading to crystallization of the film) this can result in ferroelectricity of the oxide layers. This similarity can be also seen in the connected structural and electrical properties after heavy ion radiation exposure (Figure 6). The irradiated HfZrO_2_ samples (composition Hf_0.5_Zr_0.5_O_2_) (series C) also show changes in the structural and electrical properties (Figure 6). In the XRD patterns (series C, Figure 6a), the (111) orthorhombic reflection (2θ ≈ 30.5°) is visible as the main characteristic of as-grown ferroelectric HfZrO_2_ films. Additionally, a small contribution of the monoclinic phase is present in as-grown films, which is vanishing in the XRD patterns of irradiated films at fluences above 5 × 10^11^ ions/cm^2^. Similar to results obtained for nondoped HfO_2_-based films, this monoclinic phase is likely to be transformed to a cubic phase, as described in the previous section (Figures 2 and 3). For fluences above 3 × 10^12^ ions/cm^2^, the characteristic orthorhombic (111) reflection slightly shifts toward higher 2θ diffraction angles (red dotted line), which we attribute to a possible phase transition from the orthorhombic to another crystalline phase–e.g., a tetragonal phase (*P*42/*nmc*, ICDD: 01-078-5756) or a cubic phase (like distorted *Fm*3̅*m*, ICDD: 04-011-9018). As in general, the tetragonal, cubic, and various orthorhombic phases can be hardly distinguished by XRD only, as these phases are inter-related and show very similar 2θ diffraction angles, a clear interpretation of the crystallinity cannot be made based only on XRD results. Such phenomenon is similar to the mentioned difficulty appearing for nondoped HfO_*x*_ films. As discussed above for undoped HfO_*x*_, also the shift of the reflections may be attributed to induced oxygen vacancies with a phase transition occurring for lower oxygen contents of the layer. As electrical device test, measurements of the voltage-dependent electrical polarization of the HfZrO_2_ films were recorded (Figure 6b). A correlation of the crystalline structure and electrical properties is used as a direct verification of a ferroelectric phase in the active layer. For the presented case, the HfZrO_2_-containing stacks are ferroelectric before irradiation. With increasing fluence, a degradation of the ferroelectric properties occurs evidenced by a polarization reduced from about 7.7 μC/cm^2^ (nonirradiated, as-grown reference) to 3.4 μC/cm^2^ (when irradiated with 2.4 × 10^12^ ions/cm^2^). Compared to the shift of the (111) orthorhombic reflection starting approximately at 10^12^ ions/cm^2^, changes of the polarization are already induced at lower fluences (around 5 × 10^11^ ions/cm^2^), most probably due to the creation of oxygen vacancies and other defects and not necessarily due to a phase transition (at fluences up to 5 × 10^11^ ions/cm^2^). At higher fluences, an irradiation-induced phase transition is very likely and directly connected to a drastic reduction of the electrical polarization in HfZrO_2_-based FeRAM. Combining the electrical results with the corresponding phase transition revealed by XRD, a partial transition from an orthorhombic to a nonpolar phase can be concluded. The formation of a defect-stabilized LTP cubic phase is likely, hereby, similar to the findings described for irradiated nondoped hafnium oxide films. While the irradiation damage clearly influences the polarization of the samples, it is noteworthy that additional cycling of the irradiated films (postcycling, Figure 6c) leads to a significant reopening of the polarization–voltage loops (10^4^ cycles). This is accompanied by a recovery of the polarization values (1 × 10^10^ ions/cm^2^: 11.5 μC/cm^2^; 5 × 10^11^ ions/cm^2^: 10.3 μC/cm^2^; 2.4 × 10^12^ ions/cm^2^: 7.9 μC/cm^2^). For samples irradiated at low fluences without a phase transition, a redistribution of the irradiation-induced oxygen defects is likely. At high fluences, for samples with a phase transition induced, the recovered electrical properties after postcycling (after irradiation) suggest the occurrence of a field-induced phase transformation from a nonpolar back to the ferroelectric orthorhombic phase. Interestingly, the polarization did not only get fully recovered by the cycling process, but was even further increased compared to the initial values. This improvement can be a result of the beam-induced reduction of the monoclinic phase initially present in as-grown films. Such a recovery of ferroelectric properties induced by cycling has been reported in literature for fatigued, nonirradiated PZT^69^ and HfO_*x*_-based^70^ ferroelectric films. Additionally, a comparable enhancement of the polarization in HfO_2_ thin films by light ion He bombardment was recently reported.^71^ The main difference to the presented experiments is the use of light He ions with a much lower electronic energy loss than obtained for 1.635 GeV Au ions. This is leading to a different interaction and damage process.

Further, no evidence for changes of the electrical properties and crystalline phase were found in stacks containing ferroelectric hafnium oxide-based films after proton irradiation at huge fluences of 10^15^ protons/cm^2^.^33,34^ This is not a contradiction to our observation but a direct indication that the energy loss of protons is too low to initiate a phase transition in HfZrO_2_ films. Still, material property changes, like an increase of the polarization, can be induced in HfO_2_-based ferroelectrics, which are based on nuclear interaction and ion implantation.^71^ Overall, working devices showing distinct polarization states even at rather high energies and fluences as well as polarization recovery by postcycling directly suggest that ferroelectric memories based on hafnium oxide films are radiation-hard.

### Irradiation-Induced Phase Transitions in GeSbTe-Based Phase-Change Layers and Correlated PCRAM Device Properties

**Figure 7:**
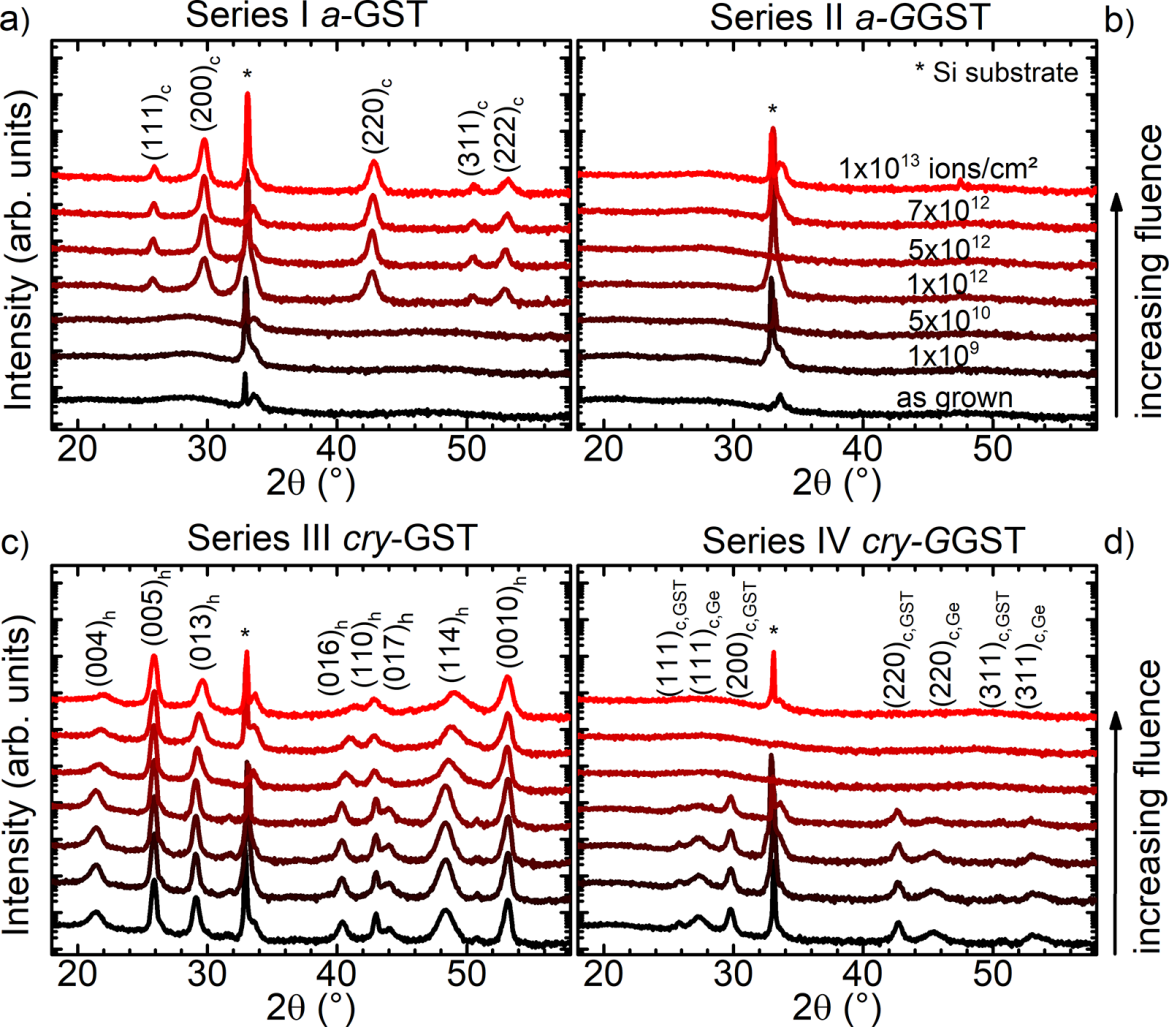
XRD patterns of as-grown and irradiated GeSbTe-based samples consisting of initially (a) amorphous Ge_2_Sb_2_Te_5_ (a-GST), (b) amorphous Ge-rich GST (a-GGST), (c) crystalline Ge_2_Sb_2_Te_5_ (cry-GST), and (d) crystalline Ge-rich GST (cry-GGST). Structural changes with increasing fluence occur for a-GST, cry-GGST, and cry-GGST films, while for a-GGST no structural change (no crystallization) can be identified. The forbidden Si (200) reflection is marked with an *. Data presented at RADECS 2020 conference; proceedings pending.^22^

While for hafnium oxide-based OxRAM and FeRAM we verified exclusive crystalline-to-crystalline phase transitions, the irradiations on phase-change PCRAM devices based on GeSbTe films discussed in this section additionally reveal different effects such as amorphous-to-crystalline and crystalline-to-amorphous transitions as well as a stable amorphous phase. A special focus of the GeSbTe-based phase-change materials is again lying on the achieved composition and initial crystallinity of the material, when comparing Ge_2_Sb_2_Te_5_ with Ge-rich GST (series I–IV in Figure 7, XRD patterns). As the phase-change memory functionality is strongly dependent on the crystallinity of the GeSbTe-based layers, induced changes can have a crucial impact on material properties and device functionality (on/off state). The initially amorphous GST (*a*-GST, series I) undergoes crystallization into a cubic phase (*Fm*3̅*m*, ICDD: 00-054-0484) at fluences of 1 × 10^12^ ions/cm^2^ and above (Figure 7 a). In contrast, there is no crystallization visible in amorphous Ge-rich GST (*a*-GGST, series II) in the XRD patterns, not even after ion exposure to the highest fluence of 1 × 10^13^ ions/cm^2^ (Figure 7b). In initially crystalline layers, the ion irradiation leads to no significant structural changes up to a fluence of 1 × 10^12^ ions/cm^2^. At higher fluences, the crystalline GST samples (*cry*-GST, series III) with a hexagonal structure (*P*3̅*m*1, trigonal, ICDD: 04-020-8161) show a slight broadening and minor shifts of the corresponding reflections (Figure 7c). In contrast, in crystalline Ge-rich samples (*cry*-GGST, series IV), a gradual decrease of the cubic reflections belonging to crystalline Ge (*Fd*3̅*m*, ICDD: 00-004-0545) and crystalline GST (representing the typical phase separation in GGST) are visible up to a fluence of 1 × 10^12^ ions/cm^2^, followed at higher fluences by a complete vanishing of these reflections in the XRD patterns (Figure 7d). In summary, all four sample series I–IV consisting of GeSbTe-based layers of different composition and initial crystallinity respond quite differently when being exposed to the same 1.635 GeV Au heavy ions of 34 keV/nm electronic energy loss.

**Figure 8:**
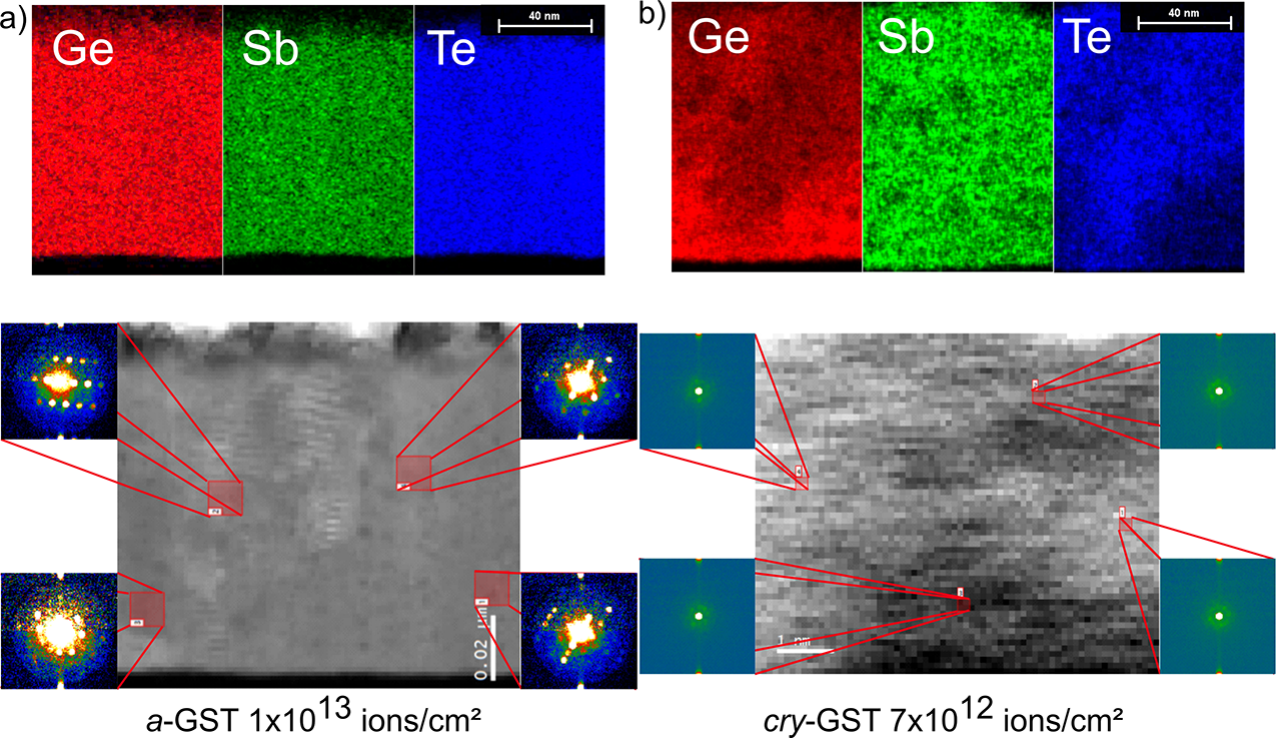
(a) EDX and HAADF-STEM images of (initially as-grown amorphous) Ge_2_Sb_2_Te_5_ (a-GST) exposed to 1 × 10^13^ ions/cm^2^, revealing the creation of a crystalline structure with large grains, which is supported by nanodiffraction patterns. (b) EDX and HAADF-STEM images of initially crystalline Ge-rich GST exposed to 7 × 10^12^ ions/cm^2^ revealing crystallites of size on the nm scale by means of few and low intensity NBED patterns. The typical Ge segregation in GGST is still visible.

Amorphous Ge-rich GST films show a higher radiation resilience than amorphous Ge_2_Sb_2_Te_5_ films, which on first glance seems to be associated with the higher temperature stability of amorphous Ge-rich GST films.^12−14^ We exclude beam-induced temperature effects during ion irradiation since the ion flux was kept below 5 × 10^8^ ions/cm^2^s. The temperature increase of the samples is estimated to be no more than 50–60 °C, which is below the crystallization and melting temperature of the GeSbTe-based materials. Nevertheless, two competing mechanisms can occur during and right after irradiation due to a temperature spike localized around the ion trajectory:^72,73^ (1) the breaking of existing bonds of the crystalline structure and (2) a temperature-induced crystallization due to a significant local temperature increase. Such thermal spikes can lead to a nucleation process, accompanied by crystallite growth and bond formation. This crystallization mechanism seems to be dominant in *a*-GST (series I), while bond-breaking dominates in the transition of *cry*-GGST to *a*-GGST (series IV). A general competition between bond-breaking and bond-(re)creation is likely, where the final result is determined by the dominating process. STEM and energy-dispersive X-ray (EDX) analysis (Figure 8), performed on selected irradiated samples of series I (1 × 10^13^ ions/cm^2^) and series IV (7 × 10^12^ ions/cm^2^), can help to better understand the described radiation-induced changes. In *a*-GST irradiated at 1 × 10^13^ ions/cm^2^ (Figure 8a), the creation of a crystalline structure with large grains is observed in the STEM images after irradiation, which is supported by nanodiffraction results. De Bastiani et al.^74^ reported a comparable crystallization revealed by Raman spectroscopy for as-grown samples, including a description of the reduction of initially Ge–Te tetrahedral bonds, which are a characteristic property of *a*-GST. Given by the overall uniform distribution of the elements Ge, Sb, and Te even after the high fluence irradiation (1 × 10^13^ ions/cm^2^) of our GST samples (EDX images of Figure 8), we assume a similar structural characteristic. From the XRD patterns as presented in Figure 7a, the crystallization of *a*-GST starts somewhere within the fluence range of 5 × 10^10^ to 1 × 10^12^ ions/cm^2^, which is quite a wide fluence spread. Additional irradiations with smaller fluence steps (not shown in Figure 7a) reveal that the phase transition from *a*-GST to the cubic GST occurs already at a fluence between 5 × 10^10^ and 3 × 10^11^ ions/cm^2^ (see Supporting Information 2, series V). This is significantly lower than the fluences necessary for the observed phase transitions of the layers of series III and IV (*cry*-GST and *cry*-GGST). Interestingly, for initial *cry*-GGST films, the structure of irradiated films (e.g., 7 × 10^12^ ions/cm^2^) seems to be amorphous, while STEM investigations revealed a preservation of the initial (characteristic) phase segregation of Ge and GeSbTe phases (Figure 8b) with presence of residual nanodiffraction patterns. Therefore, similarly to the presented results in thin hafnium oxide layers (Figures 2–4), the loss of crystalline reflections in the XRD patterns (Figure 7d) can be attributed to a grain fragmentation, which was confirmed by nanodiffraction analysis. The overall crystalline long-range order is affected after heavy ion irradiation at high fluence, still the short-range order seems to be preserved. The increased background intensity in the XRD patterns between 2θ ≈ 25° and 2θ ≈ 30° in the XRD patterns can further also be attributed to such a diffraction from nanocrystalline morphology as, e.g., reported in literature.^61,62^ Although the definition of phase-change materials includes the change between an amorphous and a crystalline state, the term “amorphous” may also relate to a nanocrystalline structure of a highly disordered nature. This nanocrystallization accompanied by a maintenance of the microcrystalline intrinsic phase segregation obtained for series IV (see STEM results) and the preserved amorphous state in XRD after irradiation of series II confirms the observed higher stability of GGST films compared to GST films, also on a microscopic scale. This is shown to be valid even after high energy heavy ion irradiation with huge fluences and turns out to be a result of the characteristic microstructure of the GGST films with a quite inhomogeneous elemental distribution of Ge, Sb, and Te (EDX in Figure 8) and the corresponding higher binding energies.^75,76^ Overall, the GGST layers tend to maintain the disordered phase as the stable phase, while this is less favorable in GST layers. It is reasonable to assume that the differences observed between the four different series I–IV are based on the different thermal conductivities of the materials, as the local heating and the heat distribution is dependent on the thermal conductivity characteristics (*a*-GST: ∼0.19 W/m*K, *cry*-GST: ∼0.57 W/m*K (cubic), and ∼1.58 W/m*K (hexagonal)^77^). The lower thermal conductivity leads to an increased temperature of the ion-induced localized thermal spike, which then results in the formation of nanocrystallites of cubic GST at high ion fluences.

**Figure 9:**
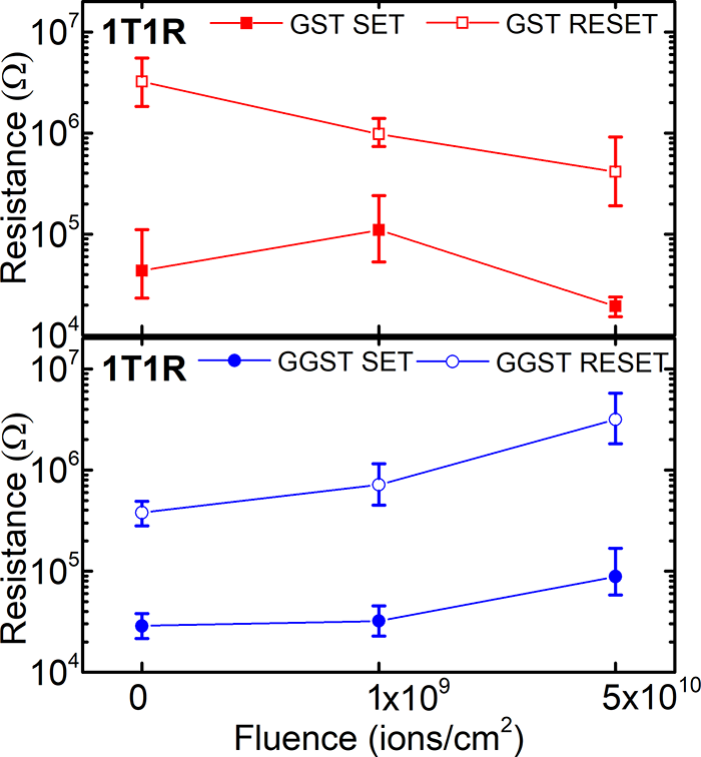
Median and ±1σ values of the device resistance of 4kb arrays in the Set and Reset state, which are based on GST (top) and on GGST (bottom) as a function of fluence. The resistances of GST-based devices decrease with increasing fluence, while for GGST the resistances increase. The memory cells are accessed by CMOS transistors. Data presented at the RADECS 2020 conference; proceedings pending.^22^

Interestingly, the observed phase changes in *cry*-GST, *a*-GGST, and *cry*-GGST occur at fluences in the range above 1 × 10^12^ ions/cm^2^ (threshold between 1 × 10^12^ ions/cm^2^ and 5 × 10^12^ ions/cm^2^), which is similar to the phase change observations made for hafnium oxide. On the one hand, the high fluence needed to introduce changes reveals the high radiation resilience of these materials. On the other hand, induced changes at high fluences can be related to overlapping ion tracks, the same phenomenon as discussed for HfO_*x*_ (above 1 × 10^12^ ions/cm^2^). Track overlapping is possibly required to induce certain phase transitions in form of a local high heat creation in GeSbTe-based films. In crystalline films, where bond breaking is likely, ion irradiation can lead to a higher (short- and long-range) disorder in the layers. Also, the 2θ-shift and the broadening of reflections in the XRD patterns (Figure 7c, series III) can be attributed to a gradual change of the out-of-plane lattice parameters after exposure to higher and higher fluences. Still, the films remain crystalline (*h*-GST), with only a slight loss of order. In amorphous films without an initial long-range order (series IV), irradiation can lead to the formation of a higher order, like medium-range or long-range ordering, possibly due to bond formation (Figure 7d). Such a crystallization becomes more probable at higher fluences, especially if a track overlapping leads to a connection of the initially separated more localized crystallization tracks. This further proves the necessity to adopt a combined, global and nanoscale, characterization effort to gain fundamental understanding of the triggered processes. Surprisingly, most reports in literature concerning irradiation of phase-change memory are lacking investigations of phase transitions. Available structural investigations are scarce and do not provide a clear picture overall. Some studies report no significant changes for light low-energy ions or protons,^28,60,61^ and even for high energy heavy ions, only small indications of a radiation-induced crystallization of amorphous GeSbTe-based layers^62^ were reported. In our study, however, irradiation experiments at considerably higher ion energy clearly provide evidence of induced microstructural changes. GeSbTe-based layers of different composition, irradiated under identical conditions, show completely different crystallinities and structural changes. These results reveal the importance of investigating the crystallinity of phase-change memory after irradiation. In particular, the observed transition from an amorphous to a crystalline GST film after irradiation at fluences between 5 × 10^10^ to 3 × 10^11^ ions/cm^2^ is exceptional, as this fluence range is much lower than observed for other transitions of GeSbTe-based materials. Therefore, a direct correlation of these findings with electrical results in GST- and GGST- based memories was performed by measurements on as-grown and irradiated state-of-the-art wall-based PCRAM devices with an electrode area of 10^–3^ μm^2^ (Figure 9). The median and ±1σ values of the device resistance in the SET and RESET state are presented in Figure 9. Overall, GGST-based PCRAM devices (1T1R) show a higher radiation resilience than GST-based devices. It should be mentioned that a solid statement about device reliability requires a larger data set with more fluence points. For fluences above 5 × 10^10^ ions/cm^2^, the access transistors in the electrical 1T1R arrays were damaged up to a full loss of addressing the transistors, which inhibited the data readout. However, by presenting and discussing the available data, likely trends of the device resistances can be given and related to possible ion-induced changes.

For GST-based devices an overall decrease of the resistances can be observed with increasing fluence. The resistance in the RESET state decreases from initially (pristine) 3 × 10^6^ Ω to 4 × 10^5^ Ω (fluence 5 × 10^10^ ions/cm^2^). At 10^9^ ions/cm^2^, the SET state in GST faces a structural relaxation, visible as an increase of the resistance, before it is decreased at 5 × 10^10^ ions/cm^2^. Also, in the SET state a decrease to 2 × 10^4^ Ω occurs after irradiation at a fluence of 5 × 10^10^ ions/cm^2^. Here, a loss of the programmed memory state is evident. For GGST-based devices, an increase of all resistance values with increasing fluence is observed (SET: 3 × 10^4^ Ω to 9 × 10^4^ Ω; RESET: 4 × 10^5^ Ω to 3 × 10^6^ Ω), while the memory window is maintained or even slightly increased. When looking for an explanation for these differences, two possible factors can be considered: (1) a possible phase transition induced by the ion irradiation and (2) detrimental effects on the CMOS access transistor functionality after ion exposure. The observed reduction of the resistance in GST-based devices at higher fluences can therefore be explained on the one hand by a possible start of a phase transition (Figure 7 and Supporting Information 2). Indeed, a fluence of 5 × 10^10^ ions/cm^2^ is already close to the fluence required for the phase transition from the amorphous to the crystalline phase observed in the XRD patterns of full-sheet GST-layers. The results are therefore in agreement with the incoming crystallization observed in the XRD patterns at higher fluence. The devices becomes more conducting with increasing fluence, which can be a result of an increasing number of localized structural changes. On the other hand, results obtained from OxRAM resistive switching have revealed that transistors are affected at 5 × 10^10^ ions/cm^2^ and 1 × 10^12^ ions/cm^2^ (increasing resistance; compare Figure 5c). As for GST-based 1T1R devices (Figure 9, red), the overall resistance is decreasing with increasing fluence, and the process seems to be dominated by an ongoing phase transition and less affected by the lowered transistor functionality. In contrast, the resistance values of both memory states (SET and RESET) are increasing with increasing fluence for GGST-based 1T1R devices (Figure 9, blue). In combination with the results observed after the exposure to 5 × 10^10^ ions/cm^2^, this gives a clear indication of a major effect on the access transistors, as there is no phase transition expected (compare Figure 7) for fluences as low as 5 × 10^10^ ions/cm^2^ or below. Ion-beam induced structural relaxation of the cubic GST and cubic Ge matrix or the formation of nanocrystalline grains by fragmentation on a local scale is possible but to a very limited extent. Due to the very small electrode area of about 10^–3^ μm^2^, the results clearly point toward a dominance of failures induced by affected CMOS transistors (single event effects). Still, the memory gap is maintained within a reasonable resistance range and the overall performance of the GGST-based devices is rated as excellent. This demonstrates the achievement of radiation-hard memory based on phase-change materials, even for the high energy and fluence values presented. In literature, mostly single event effects in electronics were investigated at low fluences, where no significant changes of the electrical properties occur.^23−26^ Single event upsets were also reported in GeSbTe-based electronic devices irradiated with 1.2 GeV Xe ions. It was assumed that the radiation induces a localized amorphization of the GST layer at the interface of the phase-change layer and the heating element.^27^ The formation of nanocrystallites/amorphization or starting crystallization can be presumed but needs to be confirmed by appropriate experiments. The presented results will be of great importance for better understanding radiation effects in PCRAM devices. This is also valid for device interactions with particles at presumably lower fluences, where phase transitions are initially not to be expected. Indeed, our results prove that electronics based on phase-change memories are affected by heavy ion irradiation and the associated induced crystallinity and microstructural changes can range from amorphous-to-crystalline, crystalline-to-crystalline, and crystalline-to-amorphous phases.

## Conclusion

Our results of the Au ion irradiation experiments reveal some common but also divergent effects occurring in hafnium oxide- and GeSbTe-based memory materials and electronics. A common property of all presented systems is that the irradiation effects strongly depend on the initial crystallinity and composition of the active layers. In highly crystalline HfO_*x*_-based materials and devices, induced oxygen defects and previously often neglected phase transitions play the major role. XRD and STEM characterization revealed a defect-induced pure crystalline-to-crystalline phase transition from the monoclinic to the LTP cubic phase of HfO_*x*_ with a significant grain fragmentation occurring in the high fluence regime. Still, electrical OxRAM devices based on crystalline HfO_2_ maintain high radiation resilience and good functionality. Our experimental approach combines nonlocal and local characterization with electrical investigations, therefore providing a test routine for future experiments on emerging nonvolatile memories. In general, FeRAM and PCRAM both strongly rely on the crystallinity and phase stability of the memory materials. In the presented FeRAM, which is also HfO_*x*_-based, oxygen vacancies are key, while in GeSbTe-based PCRAM bond-breaking and bond-(re)creation are the processes occurring under heavy ion irradiation. HfZrO_2_-based FeRAM shows the interesting property of device performance recovery by postcycling of the irradiated devices. The irradiation of amorphous and crystalline GST and Ge-rich GST films of different composition using the same irradiation conditions leads to very different responses with structural changes including amorphous-to-crystalline/crystalline-to-crystalliane and crystalline-to-amorphous phase transitions as well as a stable amorphous phase. The observed effects in GeSbTe-based materials hereby occur in a larger fluence range than in HfO_*x*_-based stacks. This result demonstrates that in particular Ge-rich GST PCM show high radiation-hardness comparable to the other memory technologies. Overall, the presented emerging nonvolatile memories are radiation-hard. By advancing structural characterization beyond global considerations down to nanoscale processes, our study provides important groundwork for investigations of analog memristive devices in future, where a multitude of resistive states representing, e.g., synaptic weights make radiation hardness even more challenging.

## Methods

### HfO_*x*_-Based Samples

For samples series A, 200 nm thick hafnium oxide films were grown at 300 °C on Si (001-oriented) substrates (with native oxide SiO_2_^78^) in a custom designed reactive molecular beam epitaxy (MBE) setup (series A) in a physical vapor deposition (PVD) process. In this growth process, hafnium (99.99% purity) metal was evaporated by using an electron beam. Oxidation is enabled by an oxygen plasma, generated using an RF plasma source at 280 W. A growth rate of 0.5 Å/s was chosen, which allowed highly controlled film growth.

For sample series B, about 10 nm thick hafnium oxide films were fabricated at 300 °C by atomic layer deposition (ALD) on TiN (∼80 nm) and SiO_2_ (∼150 nm) covered Si wafers utilizing HfCl_4_ and H_2_O precursors. The thin HfO_2_ film thickness within this stack and the usage of a TiN bottom electrode grown on a Si wafer are common industrially important stacking properties, relevant for application as memory devices.

Samples of series C contain 20 nm thin hafnium zirconium oxide (Hf_0.5_Zr_0.5_O_2_ or HfZrO_2_) films grown on TiN/SiO_2_/Si by ALD, utilizing HfCl4 and ZrCl4 precursors with H_2_O as oxidizing agent and Ar as purging gas. On top of this HfZrO_2_ layer, a TiN electrode is grown by reactive sputtering. The ferroelectric orthorhombic phase (space group *Pca*2_1_; ICDD: 04-005-5597) is achieved by a postannealing step at 400 °C for 1 h. This kind of stacking is one of the most promising material systems for industrial applications of ferroelectric memory devices.

Structural X-ray diffraction (XRD) investigations were carried out using a Rigaku SmartLab diffractometer with a rotating Cu anode (K_α_-radiation with a wavelength of 1.54 Å). To create working electronics, 10 nm thin HfO_2_ layers were integrated as OxRAM memory in a 130 nm CMOS BEOL process. The transistors are NMOS types (with silicon oxide gate) of 660 nm width and 500 nm length. To obtain electrical characteristics of memory cells irradiated with 1 × 10^12^ ions/cm^2^, a larger transistor of 6700 nm width had to be used. The TiN/Ti top electrode was achieved in a PVD process. Devices have been patterned by lithography and etching to produce cells of various diameters, in this study with a focus on 400 nm diameter memory dots for the arrays. Single devices have a device diameter of 500 nm. Electrical programming and measurements were carried out by utilizing a cascade microtech bench with an Agilent B1500 parameter analyzer. Voltage-dependent electrical polarization measurements (*P*–*V*) of the stacks containing ferroelectric HfZrO_2_ were performed using an Aixacct TF 3000 FE analyzer using a triangular waveform at a frequency of 1 kHz. Ti/Pt dots were patterned by using a shadow mask and electron beam evaporation in advance. Focused ion beam (FIB) lamella preparation was implemented using a JEOL JIB-4600F MultiBeam. In advance, 70 nm of Pt was deposited on of top the hafnium oxide layers by DC-sputtering to prevent damaging of the hafnium oxide layers during lamella preparation. Scanning transmission electron microscopy (STEM) and high-resolution transmission electron microscopy (HRTEM) images of nonirradiated reference samples (as grown) and irradiated (5 × 10^12^ ions/cm^2^) samples of series A and series B were obtained utilizing a JEOL JEM ARM-200F at 200 kV. Scanning precession electron diffraction (SPED) data sets were acquired with a Quantum Detectors MerlinEM direct electron detector (convergence angle of 5 mrad). High-angle annular dark-field (HAADF) imaging was performed at 25 mrad. Automated crystal orientation mapping (ACOM) was achieved using the ASTAR software package.^79,80^ Averaging the SPED data sets was performed via HyperSpy^81^ and OpenCV^82^ packages. Information on the python based rotational averaging process of the 4D-STEM data set as well as the raw data can be found in an open repository (TUdatalib).^66^

### GeSbTe-Based Samples

Amorphous Ge_2_Sb_2_Te_5_ samples (series I, III, and V, labeled as *a*-GST) with a thickness of 100 nm were grown at 60 °C on SiO_2_/Si in a single target sputtering process. Ge-rich GeSbTe films (series II and IV, labeled as *a*-GGST) have been grown utilizing a cosputtering process from a Ge and a GST target. Crystalline films of GST (series III, labeled as *cry*-GST) and GGST (series IV, labeled as *cry*-GGST) were achieved by postdeposition annealing of as-grown amorphous samples at 450 °C for 15 min. A 10 nm SiN encapsulation layer was deposited on top via sputtering. Electrical measurements were performed on state-of-the-art wall-based PCRAM devices embedded in the Back-End-Of-the-Line (BEOL) fabrication of 4kb arrays integrated in the memory advanced demonstrator (MAD) of CEA-Leti, which is based on 130 nm CMOS technology. TEM analyses was done on samples prepared using FIB milling utilizing a FEI dual beam Helios 450S. Each sample was protected by a platinum layer to ensure surface protection from the tails of the ion beam. A 30 kV operation voltage was used for the rough milling, followed by a reduction in the range 2–8 kV to limit surface damages. Before TEM examination, samples were cleaned with an oxygen argon plasma to remove hydrocarbon contamination. Samples were observed at 200 kV using a double-aberration-corrected FEI Titan Ultimate TEM equipped with a high-brightness electron source, a Gatan Tridiem energy filter equipped with Dual EELS and a Gatan US1000 CCD camera for diffraction patterns acquisition. The probe corrector was used to obtain a beam current of about 200 pA while maintaining nanometer resolution. The EEL spectra and diffraction scans were examined using standard tools included in the Gatan Digital Micrograph software. EDX spectrometry was done utilizing the Super X detector system. Special care was taken in the control of the electron dose to avoid damaging the Ge-rich GST alloys.

### Heavy Ion Irradiation Experiments

The irradiation experiments with 1.635 GeV Au ions were performed in two sessions at the X0-beamline at the UNILAC accelerator of the GSI Helmholtzzentrum für Schwerionenforschung in Darmstadt. All samples were irradiated under identical beam conditions. The beam flux was limited to 5 × 10^8^ ions/cm^2^ s to avoid macroscopic heating of the samples. It is estimated that the sample temperature during irradiation stayed always below 50–60 °C. Fluences of the experiments ranged from 5 × 10^9^ to 8 × 10^12^ ions/cm^2^ for HfO_*x*_-based samples and from 5 × 10^9^ to 1 × 10^13^ ions/cm^2^ for all GeSbTe-based samples. The fluence uncertainty obtained with the defocused ion beam is of the order of 10–20%. No bias was applied to electronics during irradiation. The most important parameters are summarized in Table 1.

## Supporting Information Available

XRD patterns of the 200 nm thick hafnium oxide films under STEM investigation (as grown vs 5 × 10^12^ ions/cm^2^) and rotational averages of the position averaged nanobeam electron diffraction (PANBED) patterns from the *m*-HfO_2_ and LTP *c*-HfO_2-x_ phases of the ACOM data sets; XRD patterns of as-grown and irradiated initially amorphous Ge_2_Sb_2_Te_5_ (a-GST) films of series V (PDF)
