# Supplementary material for: Structural and Electrical Response of Emerging Memories Exposed to Heavy Ion Radiation
Source: ACS Nano. 2022 Sep 16;16(9):14463–78. doi: 10.1021/acsnano.2c04841 (PMC9527794; doi:10.1021/acsnano.2c04841)
Supplement: Supplementary file 1 — nn2c04841_si_001.pdf [file nn2c04841_si_001.pdf]

Structural and electrical response of emerging memories exposed to heavy ion radiation

Tobias Vogel [1], Alexander Zintler [2], Nico Kaiser [1], Nicolas Guillaume [3], Gauthier Lefèvre [4], Maximilian Lederer [5], Anna Lisa Serra [3], Eszter Piros [1], Taewook Kim [1], Philipp Schreyer [1], Robert Winkler [2], Déspina Nasiou [2], Ricardo Revello Olivo [5], Tarek Ali [5], David Lehninger [5], Alexey Arzumanov [1], Christelle Charpin-Nicolle [3], Guillaume Bourgeois [3], Laurent Grenouillet [3], Marie-Claire Cyrille [3], Gabriele Navarro [3], Konrad Seidel [5], Thomas Kämpfe [5], Stefan Petzold [1], Christina Trautmann [6], Leopoldo Molina-Luna [2], and Lambert Alff [1]*

[1] Advanced Thin Film Technology Division, Institute of Materials Science, TU Darmstadt, Alarich-Weiss-Str. 2, 64287 Darmstadt, Germany;

[2] Advanced Electron Microscopy Division, Institute of Materials Science, TU Darmstadt, Alarich-Weiss-Str. 2, 64287 Darmstadt, Germany;

[3] CEA, LETI, Univ. Grenoble Alpes, 38000 Grenoble, France;

[4] CNRS-LTM, CEA, LETI, 38054 Grenoble, France;

[5] Fraunhofer IMPS, Center Nanoelectronic Technologies (CNT), 01109 Dresden, Germany;

[6] GSI Helmholtzzentrum fuer Schwerionenforschung, 64291 Darmstadt, Germany, and also Institute of Materials Science, TU Darmstadt, 64287 Darmstadt, Germany.

Supporting Information

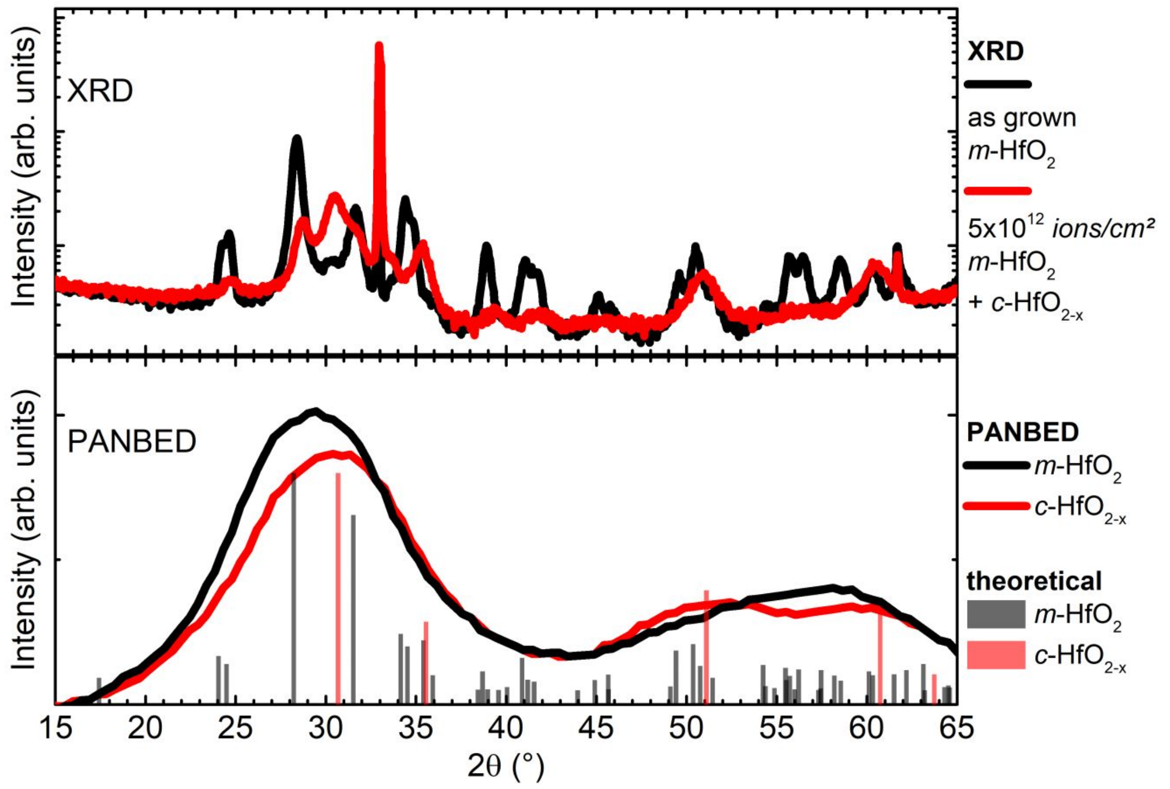

Supporting Information 1. XRD patterns of the 200 nm thick hafnium oxide films under STEM investigation (as grown vs. 5×10^{12} ions/cm²) and rotational averages of the position averaged nanobeam electron diffraction (PANBED) patterns from the *m*-HfO_2 and LTP *c*-HfO_{2-x} phases of the ACOM datasets. Simulated theoretical peak positions and intensities were deduced from VESTA. Classification of the NBED patterns was performed by template matching. By template

matching classification the two phases can clearly be separated, although the angular resolution is limited (electron probe convergence of 5 mrad).

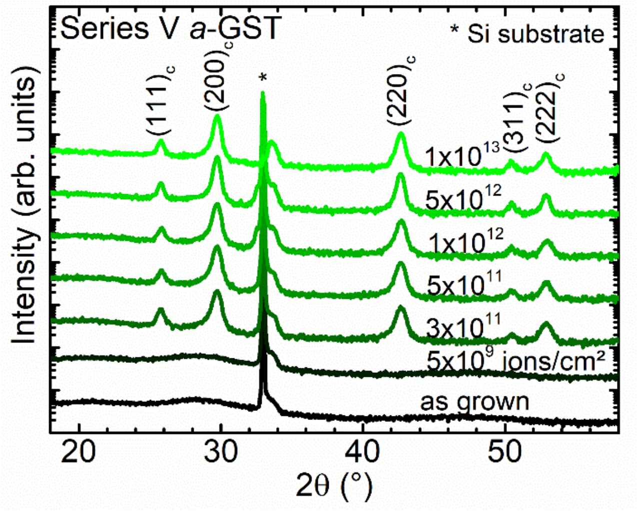

Supporting Information 2. XRD patterns of as grown and irradiated initially amorphous $Ge_2Sb_2Te_5$ (*a*-GST) films of Series V. At fluences of 3×10^{11} ions/cm² and above, the amorphous films crystallize in the cubic phase.
